# A Rare Access Site Complication after Transcatheter Aortic Valve Implantation

**Published:** 2019-10

**Authors:** Hakan Gocer, Mohammed Abusharekh, Ertugrul Ercan, Istemihan Tengiz

**Affiliations:** 1 *Department of Cardiology Medical Park Usak Hospital, Usak, Turkey.*; 2 *Department of Cardiology, Medical Park Izmir Hospital, İzmir, Turkey.*

**Keywords:** *Transcatheter aortic valve replacement*, *Intestinal perforation*, *Colectomy*

## Abstract

In the majority of patients undergoing transcatheter aortic valve implantation, the transfemoral access is the suggested approach due to its less invasive nature and feasibility in patients with suitable vascular anatomy. The complications of the transfemoral access site are generally vascular; however, we herein present a rare case of colon perforation following the transfemoral procedure owing to prior abdominal surgery. A transfemoral aortic valve was inserted on account of severe aortic stenosis and a high probability of surgical mortality. The patient developed acute abdomen following the procedure. Hemicolectomy was performed because of colonic perforation caused by femoral catheterization. The patient was well at 3 months’ follow-up.

## Introduction

Transcatheter aortic valve implantation (TAVI) is an alternative therapeutic option for severe symptomatic aortic stenosis in patients who are inoperable or have a high surgical risk. Vascular complications are documented with an incidence rate of 9.5% to even more than 50%; they are deemed potential safety limitations and associated with bleeding, transfusions, and mortality. Compared with transapical, transaxillary, or direct aortic access routes, transfemoral TAVI can be considered the least invasive choice and is hence the most widely preferred access for the procedure. Given the high burden of vascular disease in the TAVI population, preprocedural imaging such as computed tomography (CT) helps to overcome such complications by the visualization of the vascular anatomy in 3D.^1^ Although the most reported transfemoral access site adverse events are vascular, due to the anatomic proximity, intestinal complications may be rarely encountered.^2^ We herein report a rare case of subsequently surgically treated colon perforation following transfemoral TAVI in a patient with a history of abdominal surgery.

## Case Report

**Figure 1:**
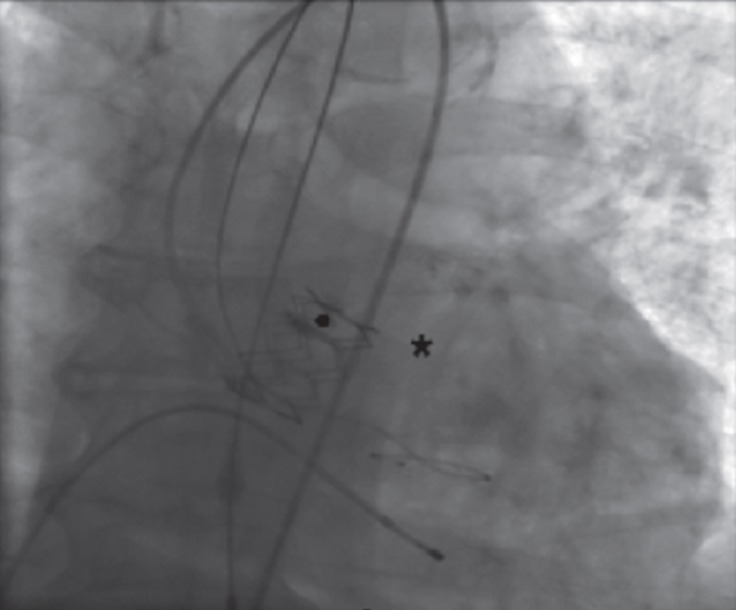
Angiographic view of transcatheter aortic valve implantation performed with a 26mm Edwards SAPIEN XT valve (star sign) implanted via the transfemoral approach for severe aortic stenosis.

An 87-year-old male patent was referred to our hospital with the complaint of exertional dyspnea. The New York Heart Association(NYHA) functional class was III on admission. The carotid arterial pulse had a delayed and plateaued peak, decreased amplitude, and gradual downslope. Cardiac auscultation revealed the characteristic crescendo-decrescendo systolic murmur of aortic stenosis. Echocardiography revealed severe aortic stenosis with a tricuspid aortic valve. The mean and peak aortic valve pressure gradient were 42 and 68 mmHg, respectively. The aortic valve area was 0.8 cm^2^. In addition, the Society of Thoracic Surgeons’ mortality score was 34.5%. The patient had a history of abdominal surgery and right iliac stenting. Because of severe pulmonary disease, he was not approved as a suitable candidate for the transapical approach; therefore, transfemoral TAVI was planned after cardiovascular surgery consultation. The procedure was performed with a 26-mm Edwards SAPIEN XT transcatheter heart valve via the transfemoral approach (Figure 1). 

Following the TAVI procedure, the mean and peak aortic valve pressure gradient decreased to 8 and 14 mmHg, respectively. There was trivial paravalvular leakage after the intervention.

**Figure 2:**
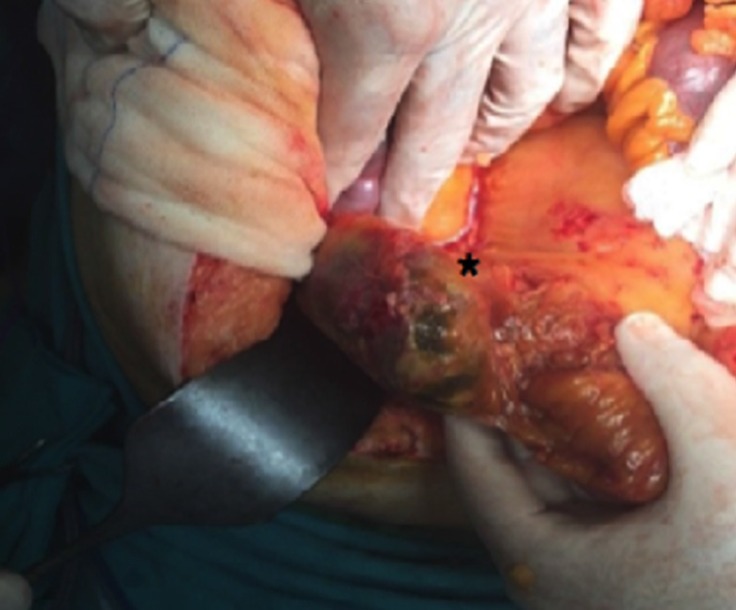
Intraoperative view of exploratory laparotomy, demonstrating colonic perforation (star sign) due to femoral catheterization

**Figure 3:**
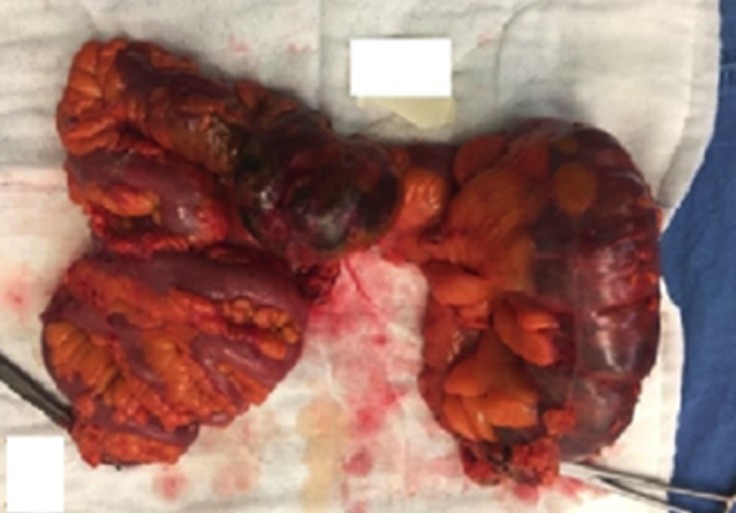
Intraoperative view of the perforated colon segment and the hemicolectomy material

After the intervention, the patient started to complain of an abrupt abdominal pain and physical examination revealed acute abdomen. Following the exclusion of mesenteric ischemia with abdominal CT angiography, the patient was urgently transferred to the general surgery department and then to the operating room. After explorative laparotomy, colon perforation due to femoral catheterization was diagnosed and hemicolectomy was performed (Figure 2 and Figure 3). The postoperative course was uneventful, and the patient was well at 3 months’ follow-up.

## Discussion

Available data on elderly candidates with an increased surgical risk reveal that TAVI is superior on the basis of mortality to standard medical therapy in prohibitive-risk patients, non-inferior or superior to surgical aortic valve replacement in high-risk patients, and non-inferior to surgery and even superior when the transfemoral approach is feasible in intermediate-risk patients. Although TAVI is considered to be a minimally invasive procedure, the periprocedural complications of the access site, mostly vascular, are associated with significantly increased patient morbidity and mortality. Despite the increased experience, improved patient selection, and the downsizing of the delivery system, the prevalence of such complications is still considered relatively high.^3^

Complications related to neighboring tissues have not been described adequately in the literature. During femoral arterial puncture technique, due to its anatomic proximity, colon perforation may rarely occur particularly in patients with a history of prior abdominal surgery. Postoperative tissue adhesions may be the probable cause of perforation. As was the case in our patient, the potential pressure necrosis associated with the previously implanted iliac stent may facilitate such complications. Relevantly, in the presence of prosthetic aortic materials, aortoenteric fistulae may develop, causing iatrogenic intestinal perforation.^4^

**Figure 4:**
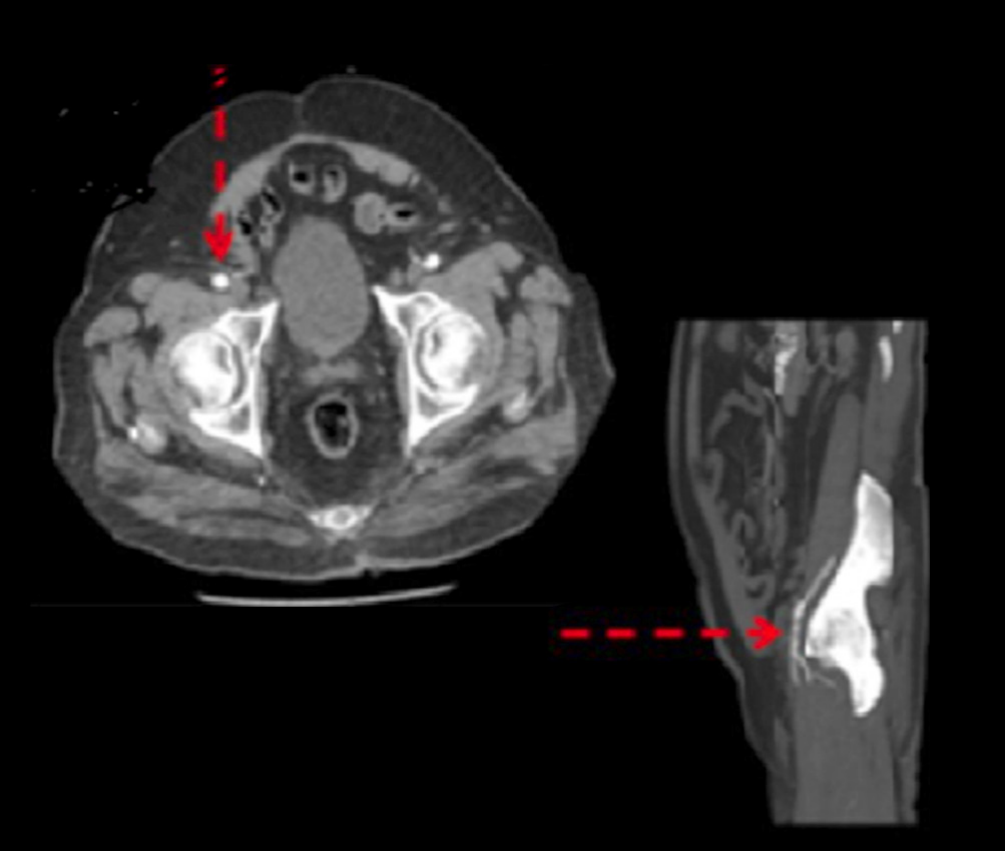
Computerized tomography angiography, indicating the close proximity of the intestinal tissue to the femoral artery access site (red arrow)

**Figure 5:**
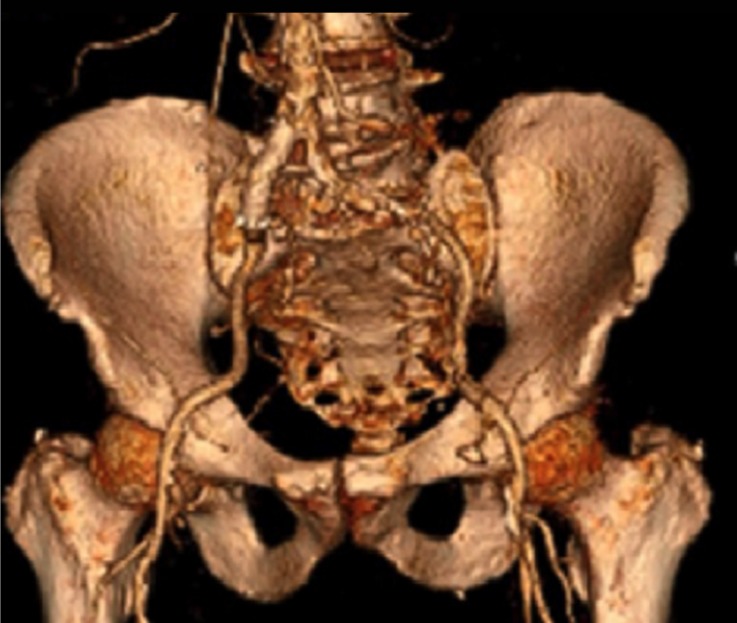
Three-dimensional reconstruction of scan images, identifying the relationship between the arteries themselves and the adjacent structures

Preprocedural imaging methods such as CT allow rapid image acquisition with high spatial resolution in 3D. In addition, not only do they confer an evaluation of the vessel size, tortuosity, extent of calcification, minimal luminal diameter, and plaque burden, but they may also detect high-risk features including dissections and complex lesions. Furthermore, it is feasible to identify the anatomy of the surrounding tissues, to accurately measure the annulus diameter and the distance between the annulus and the coronary ostia, and to determine the burden of coronary artery lesions. Preprocedural imaging was performed in our case (Figure 4 and Figure 5). 

Ultrasound guidance has been recommended to overcome the complications of the access site and to optimize the common femoral artery cannulation by reducing the number of punctures, the time required for access, bleeding, and the rates of venipuncture. On the other hand, routine use of ultrasound to guide access may not be reasonable since a recent randomized controlled trial did not show the superiority of ultrasound guidance to the anatomical landmark approach in terms of access-site outcomes at day 1 in coronary angiography and percutaneous coronary interventions.^5^

## Conclusion

Although access-site complications after transfemoral TAVI are generally vascular, complications related to the surrounding tissues such as colon perforation may rarely occur. Exhaustive preprocedural imaging using CT and ultrasound guidance for the femoral artery access appears to be helpful to overcome such complications.

## Notes:

***This paper should be cited as: ***Gocer H, Abusharekh M, Ercan E, Tengiz I. A Rare Access Site Complication after Transcatheter Aortic Valve Implantation. J Teh Univ Heart Ctr 2019;14(4):187-190.

## References

[1] Généreux P, Head SJ, Van Mieghem NM, Kodali S, Kirtane AJ, Xu K, Smith C, Serruys PW, Kappetein AP, Leon MB (2012). Clinical outcomes after transcatheter aortic valve replacement using valve academic research consortium definitions: a weighted meta-analysis of 3,519 patients from 16 studies. J Am Coll Cardiol.

[2] Fujihara S, Mori H, Kobara H, Nishiyama N, Kobayashi M, Oryu M, Masaki T (2013). An iatrogenic sigmoid perforation caused by an aortobifemoral graft mimicking an advanced colon cancer. Intern Med.

[3] Siontis GC, Praz F, Pilgrim T, Mavridis D, Verma S, Salanti G, Søndergaard L, Jüni P, Windecker S (2016). Transcatheter aortic valve implantation vs. surgical aortic valve replacement for treatment of severe aortic stenosis: a meta-analysis of randomized trials. Eur Heart J.

[4] Mack MJ, Leon MB, Smith CR, Miller DC, Moses JW, Tuzcu EM, Webb JG, Douglas PS, Anderson WN, Blackstone EH, Kodali SK, Makkar RR, Fontana GP, Kapadia S, Bavaria J, Hahn RT, Thourani VH, Babaliaros V, Pichard A, Herrmann HC, Brown DL, Williams M, Akin J, Davidson MJ, Svensson LG, PARTNER 1 trial investigators (2015). 5-year outcomes of transcatheter aortic valve replacement or surgical aortic valve replacement for high surgical risk patients with aortic stenosis (PARTNER 1): a randomised controlled trial. Lancet.

[5] Marquis-Gravel G, Tremblay-Gravel M, Lévesque J, Généreux P, Schampaert E, Palisaitis D, Doucet M, Charron T, Terriault P, Tessier P (2018). Ultrasound guidance versus anatomical landmark approach for femoral artery access in coronary angiography: a randomized controlled trial and a meta-analysis. J Interv Cardiol.

